# Protocatechuic Acid Alleviates D‐Gal‐Induced Renal Senescence and Injury in Mice by Regulating Taurine Metabolism

**DOI:** 10.1002/fsn3.71826

**Published:** 2026-05-01

**Authors:** Songhao Tian, Tao Chen, Feng Gao, Lulu Xu, Jiarui Zhao, Yuyao Du, Zhihua Zhao

**Affiliations:** ^1^ Department of Medical Laboratory Science Shanxi University of Medicine Fenyang China; ^2^ Operating Room Fenyang Hospital of Shanxi Province Fenyang China; ^3^ Graduate School Shanxi Medical University Taiyuan China; ^4^ Department of Clinical Medicine Shanxi University of Medicine Fenyang China

**Keywords:** D‐galactose, oxidative stress, protocatechuic acid, renal aging, taurine metabolism

## Abstract

Protocatechuic acid (PCA), a natural phenolic compound with antioxidant and anti‐inflammatory properties, has been proposed as a potential therapeutic agent against aging‐related diseases. This study investigated the protective effects of PCA on D‐galactose (D‐gal)‐induced renal aging in mice. PCA administration significantly improved renal function, alleviated oxidative stress and inflammatory responses, and ameliorated histopathological abnormalities. Biochemical analyses revealed reductions in serum AGEs, β‐galactosidase, creatinine, and blood urea nitrogen, accompanied by restoration of antioxidant enzyme activities and suppression of proinflammatory cytokines. Transcriptomic and metabolomic profiling further demonstrated that PCA reversed D‐gal‐induced molecular alterations, with integrated multi‐omics analysis identifying taurine and hypotaurine metabolism as the key pathway mediating its renoprotective effects. Western blot validation confirmed that PCA regulates CSAD, an enzyme essential for taurine biosynthesis. Collectively, these findings provide novel mechanistic insights into renal aging and highlight PCA as a promising natural agent for delaying kidney senescence through modulation of taurine metabolism.

## Graphical abstract

Protocatechuic acid attenuates D‐gal‐induced renal aging through regulation of CSAD‐mediated taurine metabolism, thereby alleviating oxidative stress and inflammation.

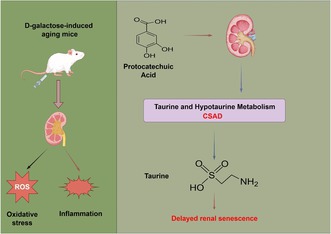

## 1 Introduction

Kidney function undergoes a gradual decline with advancing age (Herold et al. 2024). Kidney aging leads to reduced filtration function and diminished repair capacity, significantly increasing the risk of chronic kidney disease, acute kidney injury, and cardiovascular events, severely impacting the health and quality of life of the elderly (Chou and Chen 2021). The mechanisms underlying kidney aging are complex and multifaceted, involving multiple targets that interact in a cause‐and‐effect relationship. Single‐target interventions yield limited results and are prone to triggering side effects, presenting a core challenge that remains difficult to overcome (Zhang et al. 2025). Natural products offer significant advantages in treating chronic kidney disease and alleviating renal aging, including multi‐targeted intervention, improved renal function, anti‐inflammatory and antioxidant effects, high safety profiles, and dual regulation of nutrition and immunity (Josa et al. 2024). Therefore, identifying natural bioactive substances capable of delaying kidney aging and protecting renal function has become a current research focus.

High concentrations of D‐galactose (D‐gal) are metabolized by galactose oxidase into aldose and hydrogen peroxide, triggering elevated oxidative stress and mitochondrial dysfunction. This process exhibits characteristics similar to natural aging, including decreased antioxidant enzyme activity, free radical accumulation, and organ dysfunction (Zhu et al. 2023). Consequently, this model is widely employed in studies investigating aging mechanisms and anti‐aging interventions. The D‐gal model reliably reproduces lesions highly consistent with human age‐related kidney disease—including glomerular atrophy, tubular necrosis, and interstitial fibrosis—through subcutaneous or intraperitoneal injection (Li et al. 2021; Sun et al. 2025). Combining simplicity of operation, short cycle time, and reproducibility, it has become the standard tool for studying chronic kidney disease associated with aging (Shi et al. 2025; Yang et al. 2021).

Protocatechuic acid (PCA)—a natural phenolic compound abundant in green tea, acai oil, and mushrooms—exerts multifaceted effects by simultaneously activating Nrf2, inhibiting NF‐κB, and modulating MAPK and SIRT1 signaling networks (Feng et al. 2024). It integrates antioxidant, anti‐inflammatory, anti‐apoptotic, anti‐cancer, hepatoprotective, and cardiovascular‐neuroprotective activities, making it a highly promising candidate for pharmaceutical and functional food applications (Kaewmool et al. 2020; Lee and Lee 2022; Saad et al. 2025). As a natural polyphenolic metabolite, PCA demonstrates significant anti‐aging potential in the nervous system (Al Olayan et al. 2020), liver function (Tan et al. 2023), and glucose metabolism (Xiang et al. 2023) through multiple mechanisms including antioxidant, anti‐inflammatory, anti‐apoptotic effects, and regulation of energy metabolism and gut microbiota. However, its role and specific mechanisms in D‐gal‐induced renal aging remain unclear, particularly with limited in‐depth studies from a multi‐omics perspective.

This study aimed to systematically evaluate the protective effects of PCA against D‐gal‐induced renal aging and injury in mice. It further elucidated the potential mechanisms of action through a multidimensional analysis encompassing oxidative stress, inflammatory responses, histopathological alterations, and integrated transcriptomic and metabolomic profiling. The findings provide theoretical support for PCA as a potential therapeutic agent for delaying renal aging. More importantly, through multi‐omics analysis, this study first reveals the pivotal role of the taurine metabolic pathway in PCA‐mediated renal protection, offering new perspectives and therapeutic targets for understanding the mechanisms of renal aging and developing prevention and treatment strategies.

## 2 Materials and Methods

### 2.1 Chemicals and Reagents

PCA (purity≥ 99%) was obtained from Hangzhou Weibolei Biotechnology Co. Ltd. (Hangzhou, China). D‐gal was purchased from Beijing Solarbio Science & Technology Co. Ltd. (Beijing, China). Assay kits for malondialdehyde (MDA), superoxide dismutase (SOD), glutathione peroxidase (GPx), creatinine (Cr), and blood urea nitrogen (BUN) were also supplied by Beijing Solarbio Science & Technology Co. Ltd. (Beijing, China). Enzyme‐linked immunosorbent assay (ELISA) kits for advanced glycation end‐products (AGEs), β‐galactosidase, tumor necrosis factor‐α (TNF‐α), interleukin‐6 (IL‐6), and interleukin‐1β (IL‐1β) were purchased from Xiamen Lunchangshuo Biotechnology Co. Ltd. (Xiamen, China). The rabbit anti‐β‐actin antibody (l102) was obtained from Bioworld Technology Inc. (Nanjing, China). Rabbit anti‐CSAD antibody (A13845) was purchased from ABclonal Technology Co. Ltd. (Wuhan, China). HRP‐conjugated AffiniPure goat anti‐rabbit IgG (H + L) (BA1054) was purchased from Wuhan Boster Bioengineering Co. Ltd. (Wuhan, China). All other chemicals used in this study were of analytical grade.

### 2.2 Experimental Animals and Grouping

Healthy male BALB/c mice (8 weeks old) were purchased from Sipeifu Biotechnology Co. Ltd. (Beijing, China). All animals were housed under specific pathogen‐free (SPF) conditions with controlled temperature (22°C ± 2°C), humidity (55% ± 5%), and a 12 h light/dark cycle, with free access to food and water. All animal procedures were approved by the Animal Ethics Committee of Fenyang College of Shanxi Medical University (Approval No. 2025043) and conducted in accordance with institutional and national guidelines for animal care and use. Mice were randomly assigned to five groups (*n* = 8 per group) as follows: Control group (Control); D‐gal model group (D‐gal, 400 mg/Kg, intraperitoneal injection); D‐gal + low‐dose PCA group (PCA‐L + D‐gal, PCA, 50 mg/Kg, intramuscular injection); D‐gal + high‐dose PCA group (PCA‐H + D‐gal, PCA, 100 mg/Kg, intramuscular injection); PCA high‐dose group (PCA‐H, PCA, 100 mg/kg, intramuscular injection). The dosage and duration of D‐gal administration for inducing renal aging were determined according to the method described by Yang et al. and lasted for 10 weeks (Yang et al. 2021). The PCA dosage and treatment regimen were adopted from Bai et al. (2023), with PCA intramuscularly administered for 9 consecutive days starting 9 days before the end of the D‐gal induction period. Body weight was recorded weekly. At the end of treatment, mice were anesthetized, and blood samples were collected from the retro‐orbital venous plexus. Kidneys were excised, weighed, and either fixed in 4% paraformaldehyde or snap‐frozen in liquid nitrogen for subsequent analyses. The kidney index was calculated as kidney weight/body weight × 100%.

### 2.3 Serum and Renal Tissue Index Detection

At the end of the experiment, mice were fasted for 12 h, anesthetized, and sacrificed for the collection of blood and kidney samples. Serum was separated by centrifugation at 3000 rpm for 15 min, and biochemical parameters were determined using the corresponding commercial assay kits according to the manufacturer's instructions. Kidney tissues were homogenized on ice, and inflammatory and antioxidant indicators were measured following the instructions of the respective kits. Portions of renal tissue were fixed for histopathological examination, while the remaining samples were stored at −80°C for subsequent analyses. Serum Cr and BUN levels were measured using an automatic biochemical analyzer.

### 2.4 Histological Analysis

Kidney tissues were fixed in 4% paraformaldehyde for 24 h, dehydrated, and embedded in paraffin using standard procedures. Sections with a thickness of 5 μm were prepared and stained with hematoxylin and eosin (HE). Morphological changes in glomeruli and renal tubules were examined under a light microscope.

### 2.5 Western Blot Analysis

Western blot analysis was performed to determine the expression levels of target proteins in kidney tissues. Briefly, kidney tissues were homogenized on ice using a tissue homogenizer and lysed in RIPA buffer containing protease and phosphatase inhibitors. The lysates were centrifuged at 12,000 rpm for 10 min at 4°C, and the supernatants were collected as total protein extracts. Protein samples were denatured at 100°C for 10 min, and protein concentrations were determined using a protein quantification kit. Equal amounts of protein were mixed with loading buffer, separated by SDS‐PAGE, and subsequently transferred onto PVDF membranes.

After blocking with 5% blocking buffer, the membranes were washed with TBST and incubated overnight at 4°C with primary antibodies diluted in TBST. The membranes were then washed with TBST and incubated with HRP‐conjugated secondary antibodies for 1 h at room temperature. After further washing, protein bands were visualized using an enhanced chemiluminescence (ECL) detection system and captured with a chemiluminescence imaging system. β‐actin was used as the loading control. Band intensities were quantified using ImageJ software to evaluate the relative expression levels of the target proteins.

### 2.6 Transcriptomics Analysis

Kidney tissues were collected from the PCA‐H + D‐gal group for transcriptomic and metabolomic analyses, and the experimental results were denoted as the PCA group. Total RNA was extracted using TRIzol (Invitrogen) and assessed for quality and integrity (NanoDrop ND‐1000, Agilent 2100 Bioanalyzer). Samples meeting RNA concentration > 50 ng/μL and RIN > 7 were used for library construction. Poly (A) mRNA was enriched with Dynabeads Oligo (dT) (Thermo Fisher), fragmented, and reverse‐transcribed into cDNA. Libraries (~300 bp) were prepared following standard protocols and sequenced on an Illumina Novaseq 6000 platform.

Raw reads were processed with fastp and aligned to the *Mus musculus* reference genome (GRCm39) using HISAT2. Transcript assembly and quantification (FPKM) were performed with StringTie. Differentially expressed genes were identified using edgeR (fold change > 2 or < 0.5, *p* < 0.05), and functional enrichment (GO and KEGG) was analyzed with DAVID.

### 2.7 Metabolomics Analysis

Kidney tissues were extracted with prechilled 50% methanol, and supernatants were collected after protein precipitation. QC samples were prepared by pooling aliquots from all extracts.

Metabolomic profiling was performed on a TripleTOF 5600 Plus mass spectrometer (SCIEX) in both positive and negative ion modes, coupled to an ACQUITY UPLC T3 column (100 mm × 2.1 mm, 1.8 μm, Waters). MS data were acquired in information‐dependent acquisition (IDA) mode over an m/z range of 60–1200.

Raw data were converted to mzXML format and processed in R using the XCMS, CAMERA, and metaX packages to generate a feature matrix containing retention time, m/z, and peak intensity. Metabolites were annotated against the KEGG and HMDB databases with a mass tolerance of 10 ppm. Features detected in < 50% of QC samples or < 80% of biological samples were removed, and missing values were imputed using the k‐nearest neighbor (KNN) method. Data were normalized using the probabilistic quotient normalization (PQN) algorithm and corrected for batch effects using QC‐robust spline correction. Principal component analysis was used to evaluate data quality and detect potential outliers.

Differential metabolites were identified using Student's *t*‐test with false discovery rate (FDR) correction (Benjamini‐Hochberg). Supervised partial least squares discriminant analysis (PLS‐DA) was performed using metaX to identify discriminative metabolic features, and variables with a variable importance in projection (VIP) value > 1.0 were considered significant contributors to group separation.

### 2.8 Statistical Analysis

All experimental data are presented as the mean ± SEM. Statistical analyses were performed using SPSS software (version 22.0; IBM, Armonk, NY, USA). Comparisons between the Control and D‐gal groups were conducted using Student's *t*‐test. Differences among PCA treatment groups and the D‐gal group were evaluated by one‐way analysis of variance (ANOVA), followed by Dunnett's post hoc test. A value of *p* < 0.05 was considered statistically significant.

## 3 Results

### 3.1 PCA Alleviates D‐Gal‐Induced Renal Senescence and Injury in Mice

**FIGURE 1:**
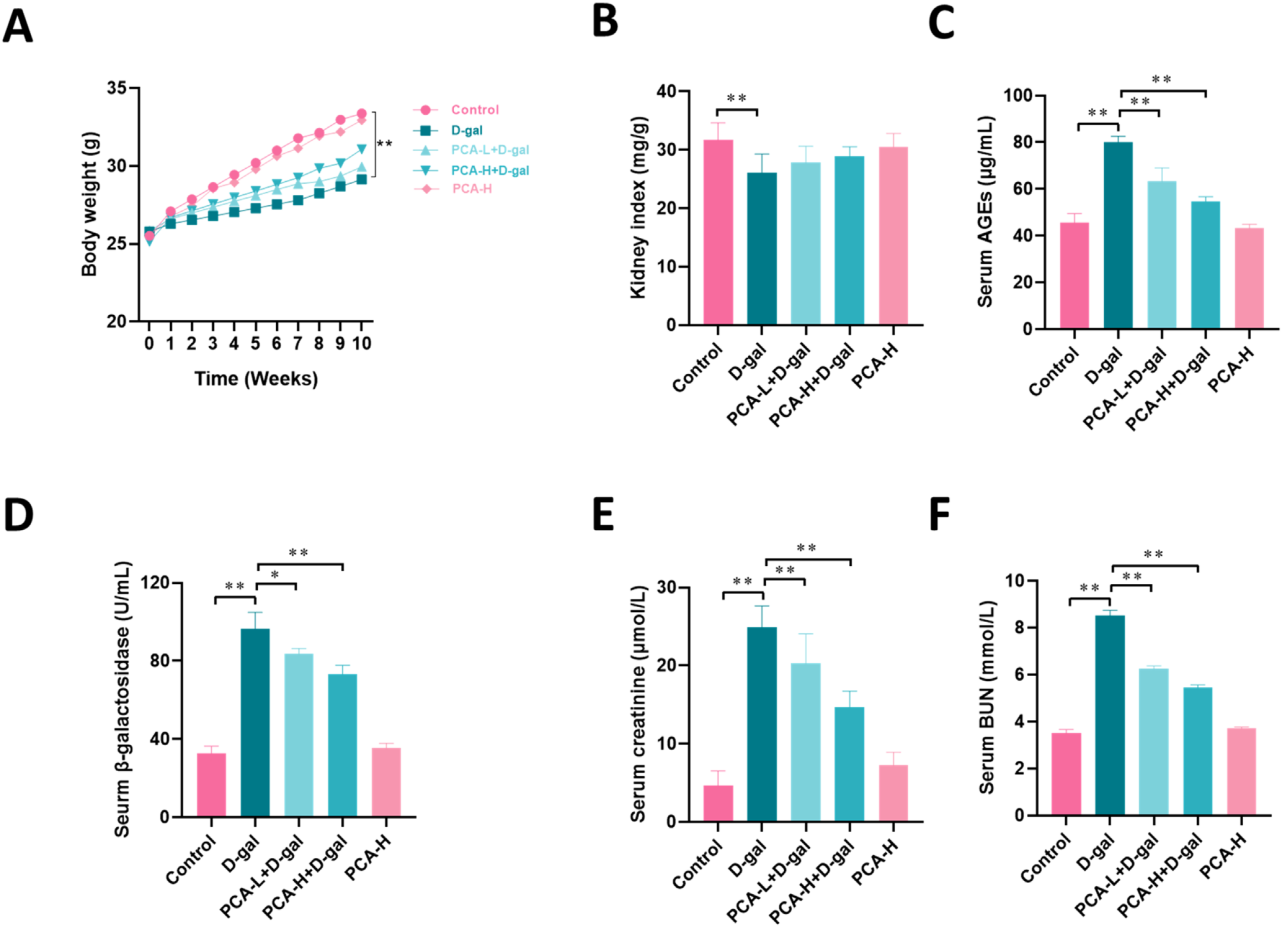
PCA alleviates D‐gal‐induced aging and renal injury in mice. (A) Body weight gain curve. (B) Kidney index. (C) Serum AGEs level. (D) Serum β‐galactosidase activity. (E) Serum creatinine level. (F) Blood urea nitrogen (BUN) level. Data are presented as mean ± SEM. *p < 0.05, **p < 0.01.

As shown in Figure 1, compared with the control group, mice in the D‐gal group exhibited a significantly slower increase in body weight (*p* < 0.01, Figure 1A) and a markedly reduced kidney index (*p* < 0.01, Figure 1B). Meanwhile, serum levels of AGEs (Figure 1C), β‐galactosidase (Figure 1D), Cr (Figure 1E), and BUN (Figure 1F) were all significantly elevated (*p* < 0.01), indicating that D‐gal successfully induced renal senescence and injury in mice. In contrast, PCA intervention markedly ameliorated these abnormalities. Specifically, PCA treatment promoted body weight gain (Figure 1A), partially restored the kidney index (Figure 1B), and significantly decreased serum AGEs, β‐galactosidase, Cr, and BUN levels (*p* < 0.01, Figure 1C–F). Moreover, the protective effect was more pronounced in the high‐dose PCA group, with values approaching those of the control group. Collectively, these results demonstrate that PCA effectively attenuates D‐gal‐induced renal senescence and injury in mice.

### 3.2 PCA Alleviates Oxidative Stress and Inflammation in D‐Gal‐Induced Mice Kidneys

**FIGURE 2:**
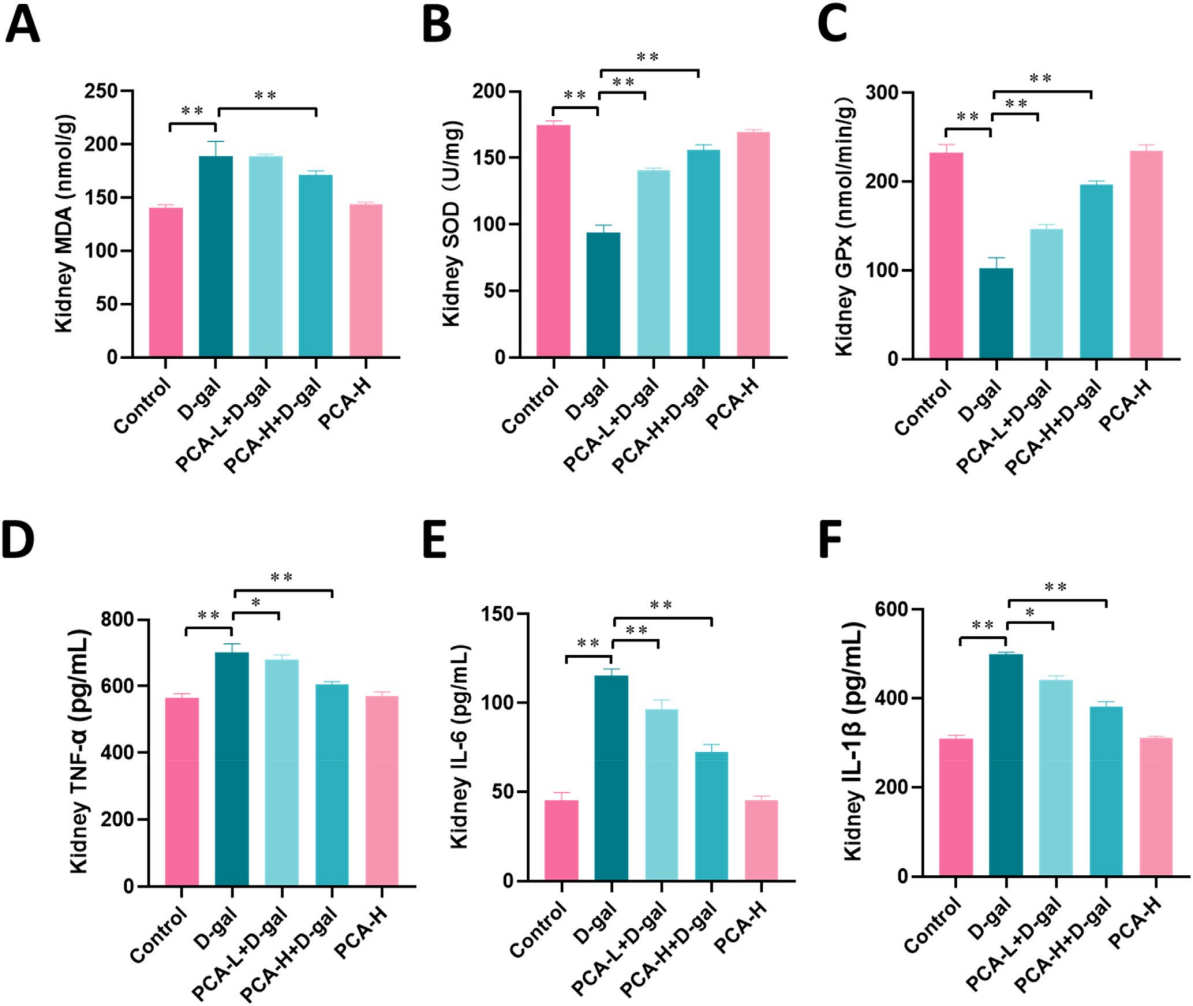
PCA reduces renal oxidative stress and inflammation in D‐gal‐treated mice. (A) Renal MDA content. (B) Renal SOD activity. (C) Renal GPx activity. (D) Renal TNF‐α level. (E) Renal IL‐6 level. (F) Renal IL‐1β level. Data are presented as mean ± SEM. *p < 0.05, **p < 0.01.

As shown in Figure 2, compared with the control group, mice in the D‐gal group exhibited significantly increased renal MDA levels (*p* < 0.01, Figure 2A), whereas SOD and GPx activities were markedly decreased (*p* < 0.01, Figure 2B,C), indicating pronounced oxidative stress. Meanwhile, renal levels of TNF‐α, IL‐6, and IL‐1β were significantly elevated in the D‐gal group (*p* < 0.01, Figure 2D–F), reflecting an enhanced inflammatory response. In contrast, PCA administration significantly ameliorated these abnormalities by reducing MDA levels, restoring SOD and GPx activities (*p* < 0.01, Figure 2A–C), and suppressing the excessive production of TNF‐α, IL‐6, and IL‐1β (*p* < 0.05, Figure 2D–F). Moreover, the protective effects were more pronounced in the high‐dose PCA group, with values approaching those of the control group. Collectively, these results indicate that PCA protects against D‐gal‐induced renal injury by alleviating oxidative stress and inflammation.

### 3.3 PCA Alleviated D‐Gal‐Induced Histological Damage in Mice Kidneys

**FIGURE 3:**
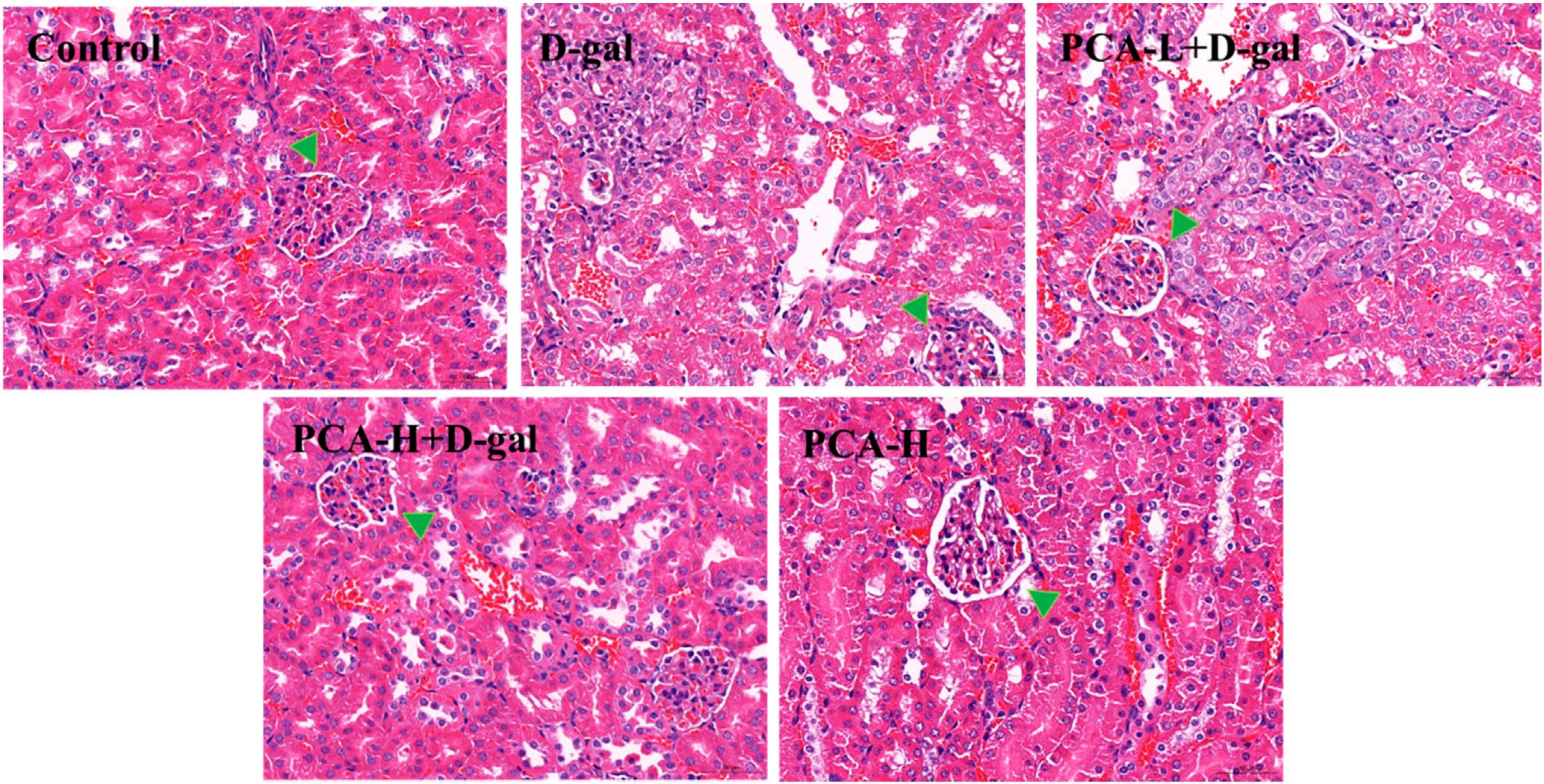
PCA attenuates D‐gal‐induced histopathological damage in mouse kidneys. Representative HE staining images (400×). Green arrows indicate glomeruli.

Further examination of the effect of PCA on mouse kidneys was conducted with HE staining (Figure 3). Results revealed that in the control group, glomeruli appeared round, tubules were neatly arranged with distinct lumens, and no significant pathological changes were observed. In the D‐gal group, glomerular morphology was altered with signs of atrophy, blurred tubular boundaries, and inflammatory cell infiltration. Treatment with various concentrations of PCA effectively ameliorated these symptoms, indicating that PCA provides significant protective effects against D‐gal‐induced renal injury.

### 3.4 Effects of PCA on the Transcriptome of D‐Gal‐Induced in Mice Kidneys

**FIGURE 4:**
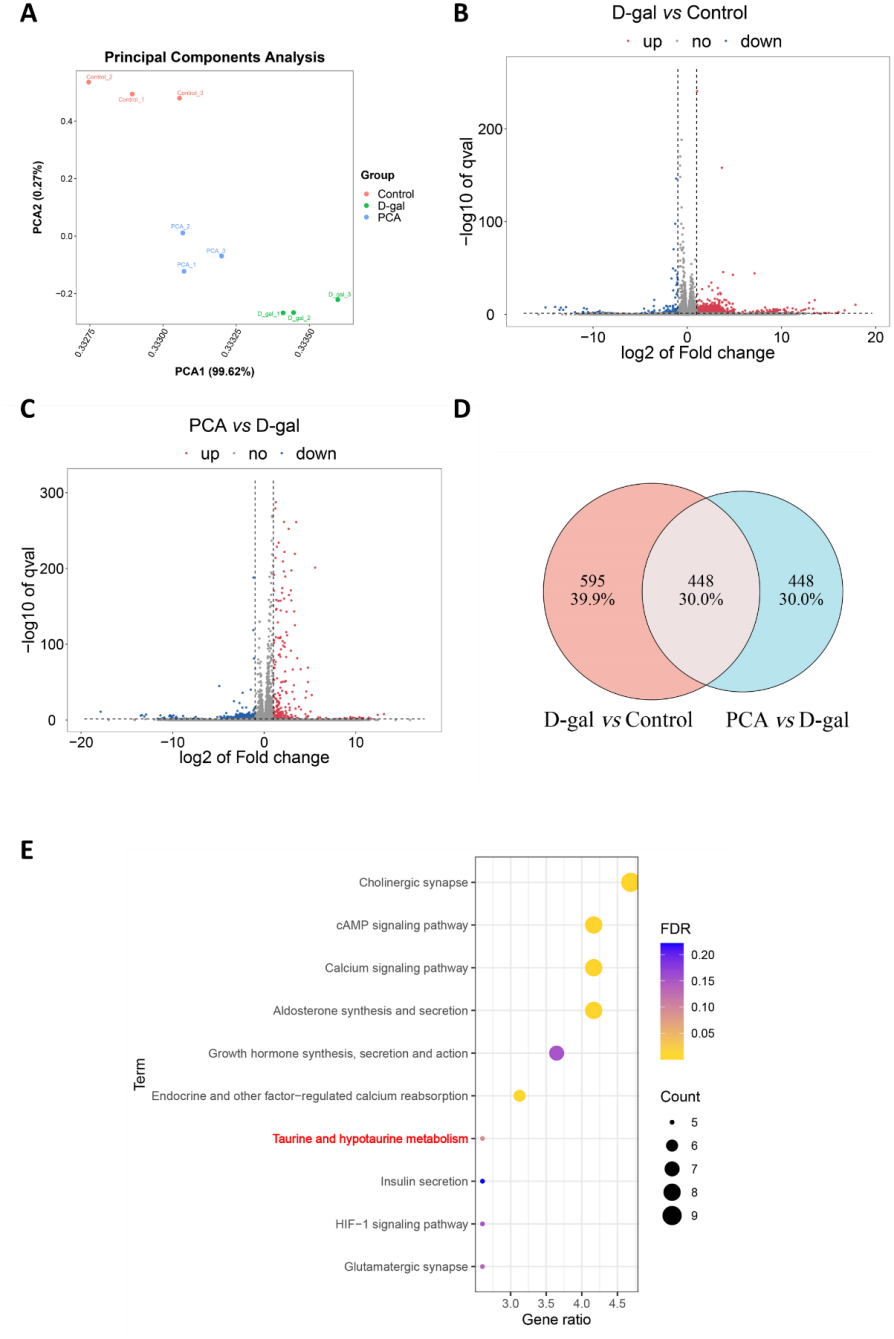
Transcriptomic analysis of renal tissues following PCA treatment. (A) Principal component analysis. (B) Volcano plot of differentially expressed genes (DEGs) between control and D‐gal groups. (C) Volcano plot of DEGs between PCA and D‐gal groups. (D) Venn diagram showing common DEGs among the three groups. (E) KEGG enrichment analysis of the common DEGs.

Transcriptomic analysis further examined the effects of PCA on gene expression in mice kidneys. Principal component analysis revealed significant differences in gene expression among the Control, D‐gal, and PCA groups (Figure 4A). The mechanism of PCA action was analyzed by comparing gene expression across these groups. Compared to the Control group, the D‐gal group exhibited up‐regulation of 862 differentially expressed genes and down‐regulation of 181 (Figure 4B). Compared to the D‐gal group, the PCA group showed up‐regulation of 302 differentially expressed genes and down‐regulation of 594 (Figure 4C). Venn analysis identified 448 differentially expressed genes common to all three groups (Figure 4D). These 448 differentially expressed genes may be closely associated with the alleviation of D‐gal‐induced renal injury by PCA. Consequently, KEGG analysis of these genes revealed enrichment in pathways including the cholinergic synapse, cAMP signaling pathway, calcium signaling pathway, and taurine and hypotaurine metabolism, suggesting PCA may exert its protective effects through these pathways (Figure 4E).

### 3.5 Effects of PCA on the Renal Metabolome of D‐Gal‐Induced Mice

**FIGURE 5:**
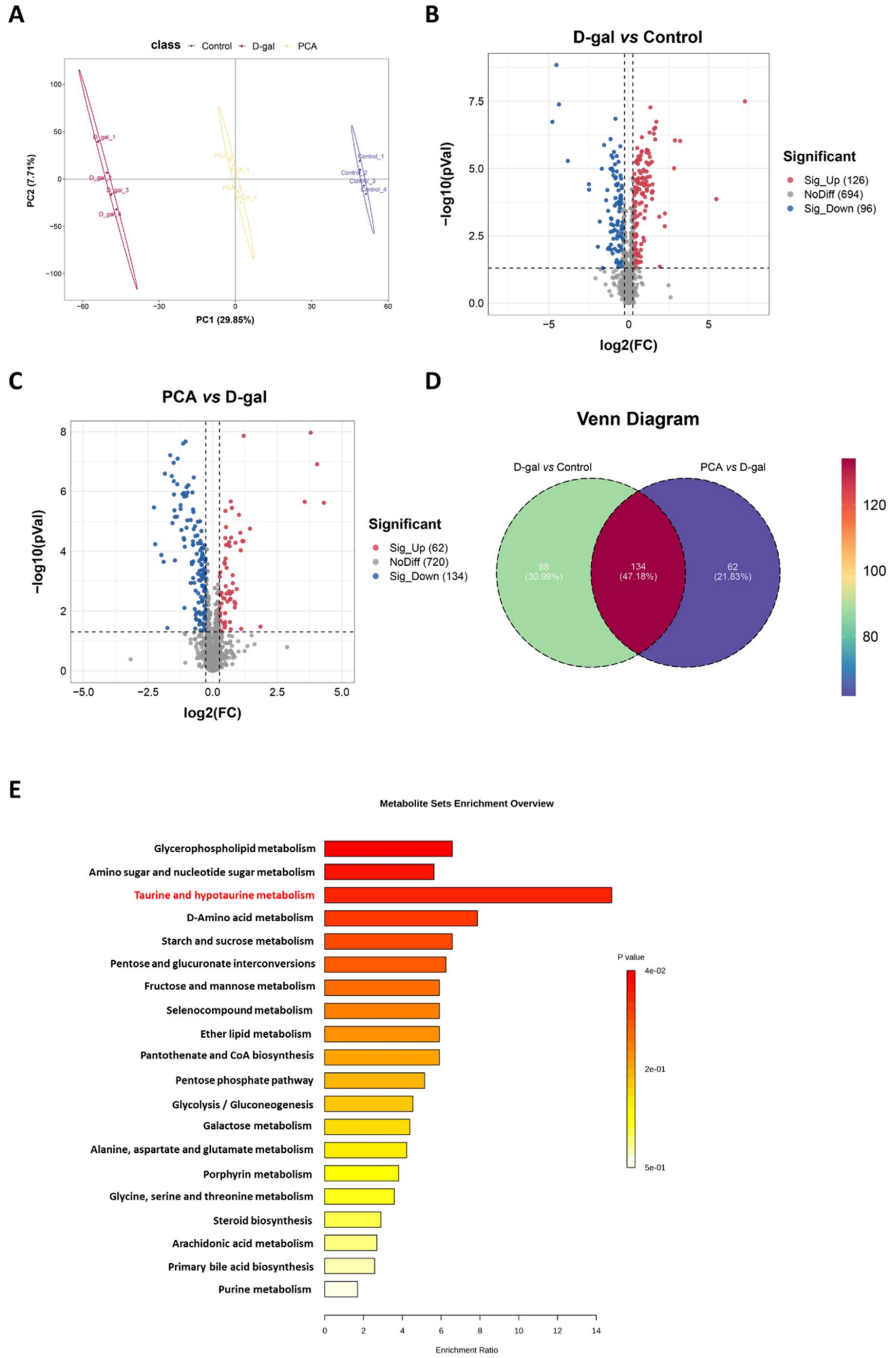
Metabolomic profiling of renal tissues following PCA treatment. (A) PLS‐DA analysis. (B) Volcano plot of differential metabolites between control and D‐gal groups. (C) Volcano plot of differential metabolites between PCA and D‐gal groups. (D) Venn diagram of shared differential metabolites among the three groups. (E) KEGG enrichment analysis of the shared differential metabolites.

Metabolomics‐based investigation of the effects of PCA on renal metabolome profiles in D‐gal‐induced mouse. PLS‐DA analysis revealed significant differences in metabolome profiles among the Control, D‐gal, and PCA groups (Figure 5A). Volcano plots illustrated the distribution of differentially expressed metabolites across groups. Compared to the Control group, the D‐gal group exhibited 126 upregulated and 96 downregulated metabolites (Figure 5B). Compared to the D‐gal group, the PCA group showed 62 upregulated and 134 downregulated metabolites (Figure 5C). Venn analysis identified 134 differentially expressed metabolites common to all three groups (Figure 5D). These metabolites may be regulated by PCA. KEGG analysis revealed that these metabolites are associated with Glycerophospholipid metabolism, Amino sugar and nucleotide sugar metabolism, and Taurine and hypotaurine metabolism (Figure 5E).

### 3.6 PCA Protects D‐Gal‐Induced Renal Injury in Mice by Modulating Taurine and Hypotaurine Metabolism

**FIGURE 6:**
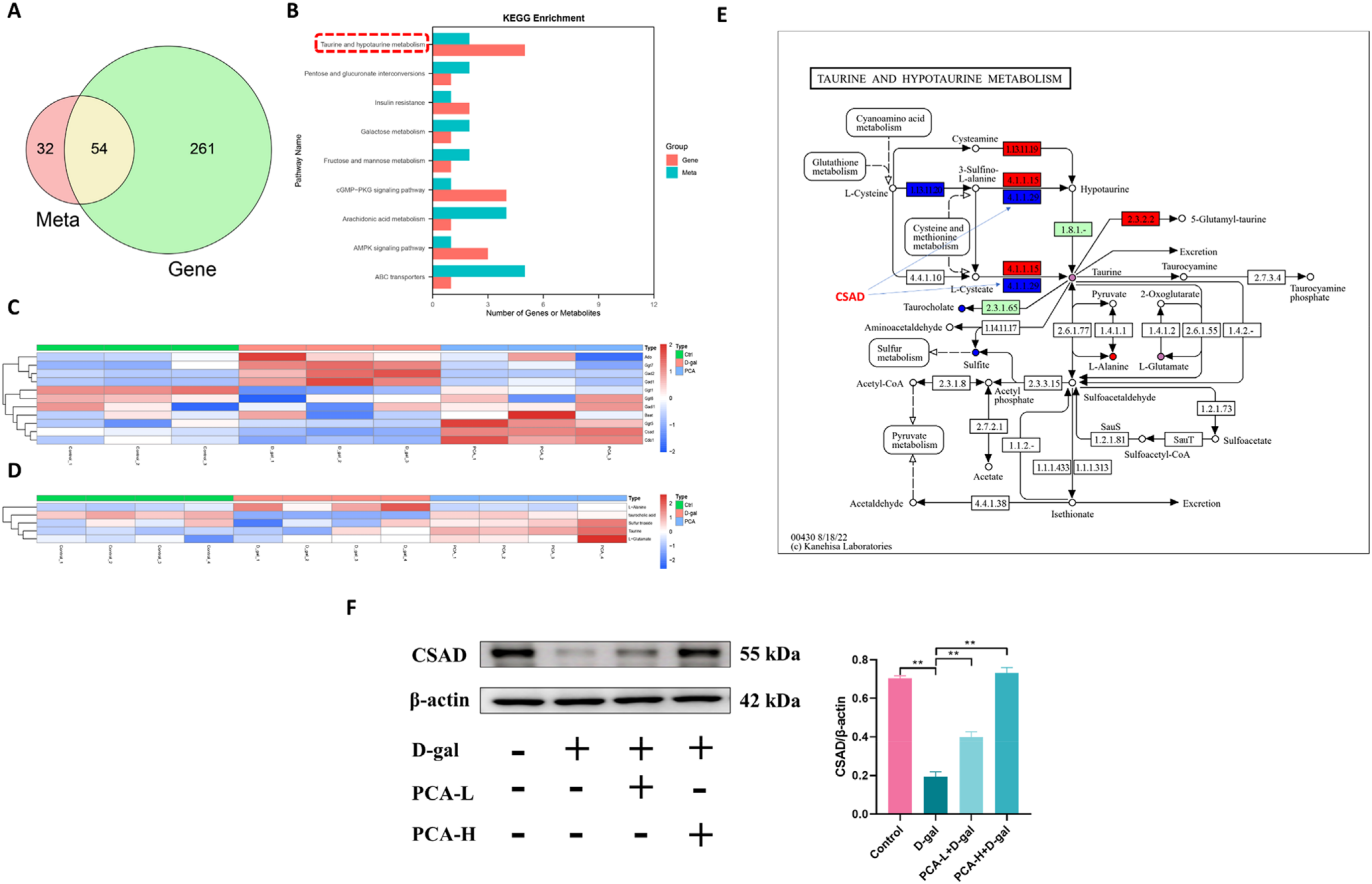
Integrated transcriptomic and metabolomic analysis reveals that PCA alleviates renal injury by modulating taurine metabolism. (A) Venn diagram of KEGG pathways shared by differential genes and metabolites among the three groups. (B) Top 10 pathways with the highest number of shared DEGs and metabolites. (C) Heatmap of relative expression of DEGs in taurine and hypotaurine metabolism. (D) Heatmap of relative abundance of differential metabolites in taurine and hypotaurine metabolism. (E) Schematic representation of taurine and hypotaurine metabolism, with upregulated DEGs or metabolites in the D‐gal group (vs. Control) that were downregulated by PCA treatment shown in red, downregulated DEGs or metabolites in the D‐gal group that were upregulated by PCA treatment shown in blue, and unaltered DEGs or metabolites shown in purple. (F) Western blot validation of the taurine metabolism‐related enzyme identified in panel E. Data are presented as mean ± SEM. **p < 0.01.

Integration of metabolome and transcriptome data reveals potential mechanisms by which PCA alleviates D‐gal‐induced renal injury in mice. Venn analysis identified 54 overlapping pathways among the common differential metabolites and differentially expressed genes in the KEGG enrichment pathways across the Control, D‐gal, and PCA groups (Figure 6A). Among these 54 pathways, the “Taurine and hypotaurine metabolism” pathway ranked first in terms of the combined number of differentially expressed genes and differential metabolites it contained (Figure 6B). Therefore, it is speculated that taurine and hypotaurine metabolism plays a primary role in the alleviation of D‐gal‐induced renal injury by PCA. Heatmaps displayed the relative abundance of differentially expressed genes (Figure 6C) and metabolites (Figure 6D) within the taurine and hypotaurine metabolism pathway across groups. Results indicate PCA effectively modulates the expression of these differentially expressed genes and metabolites. Mapping these metabolites and genes onto the Taurine and hypotaurine metabolism pathway reveals their close association with taurine metabolism (Figure 6E). To further validate this hypothesis, the expression of CSAD, a key enzyme involved in taurine metabolism, was detected by Western blotting. The results showed that, consistent with the trend of differentially expressed genes (Figure 6C), PCA significantly reversed the D‐gal‐induced downregulation of CSAD (*p* < 0.01) (Figure 6F). These findings suggest that PCA may exert its renal protective effects by influencing taurine‐related metabolism in the kidneys.

## 4 Discussion

In this study, we demonstrated that PCA, a naturally occurring phenolic compound, exerts significant protective effects against D‐gal‐induced renal aging and injury in mice. PCA administration effectively improved renal function, alleviated oxidative stress and inflammatory responses, and attenuated histopathological abnormalities. More importantly, through integrative transcriptomic and metabolomic analyses, we identified the taurine and hypotaurine metabolism pathway as a key mediator of the renoprotective effects of PCA. These findings provide novel insights into the mechanisms of renal aging and highlight PCA as a promising therapeutic candidate for delaying kidney senescence.

Kidney aging is characterized by a decline in filtration function, impaired repair capacity, and increased susceptibility to chronic kidney disease and acute kidney injury (Yamamoto and Isaka 2024). The D‐gal‐induced aging model has been widely used to mimic these pathological changes, as excessive D‐gal promotes oxidative stress, mitochondrial dysfunction, and inflammatory responses, which recapitulate features of natural aging (Liao et al. 2023; Ma et al. 2024; Yin et al. 2025). Consistent with previous reports, we observed significant increases in serum AGEs, β‐galactosidase, Cr, and BUN levels, as well as glomerular atrophy, tubular injury, and inflammatory infiltration in D‐gal‐treated mice. PCA supplementation significantly reversed these alterations, suggesting its strong potential to mitigate renal aging.

Oxidative stress and inflammation are recognized as two central drivers of renal aging (Ebert et al. 2021; Yamamoto and Isaka 2024). Excessive reactive oxygen species (ROS) production impairs mitochondrial function, damages DNA, proteins, and lipids, and ultimately accelerates cellular senescence (Martini and Passos 2023; Shmulevich and Krizhanovsky 2021). Meanwhile, chronic activation of inflammatory pathways further exacerbates kidney injury (Yeh et al. 2024). Our results showed that PCA significantly decreased renal MDA levels while restoring SOD and GPx activities, indicating potent antioxidant capacity. Moreover, PCA suppressed the overproduction of TNF‐α, IL‐6, and IL‐1β, thereby alleviating the inflammatory burden. These findings are consistent with previous studies showing that PCA exerts antioxidant and anti‐inflammatory effects through Nrf2 activation and NF‐κB inhibition (Li et al. 2023; Xiao et al. 2021). Taken together, PCA provides dual protection by counteracting both oxidative stress and inflammation during renal aging.

Histological observations further confirmed the protective role of PCA. D‐gal administration led to glomerular shrinkage, tubular degeneration, and inflammatory infiltration, which are typical pathological features of renal senescence (Chi et al. 2022; Zhang et al. 2023). PCA treatment effectively alleviated these lesions, with high‐dose administration showing the most pronounced effects. These morphological improvements parallel the biochemical findings and strengthen the evidence for PCA's renoprotective role.

To further elucidate the molecular basis of PCA's effects, we performed transcriptomic and metabolomic analyses. Transcriptome profiling revealed that PCA reversed a large subset of D‐gal‐induced differentially expressed genes, particularly those enriched in signaling pathways related to oxidative stress, inflammation, and energy metabolism. Among them, taurine and hypotaurine metabolism emerged as a prominent pathway. Metabolomic profiling supported this finding, showing that PCA significantly modulated taurine‐related metabolites. Integration of multi‐omics data confirmed that the taurine and hypotaurine metabolism pathway had the highest number of overlapping differentially expressed genes and metabolites, suggesting it plays a pivotal role in mediating PCA's renoprotective effects.

Taurine is a sulfur‐containing amino acid widely distributed throughout multiple organs in the human body, such as the heart, eyes, liver, and kidneys (Rais et al. 2023). As we age, taurine levels in the blood decrease significantly, a process closely associated with the aging process (Singh et al. 2023). Research indicates that taurine deficiency may be a driving factor in aging. Supplementing with taurine can slow down aging characteristics and reduce the risk of age‐related diseases (Santulli et al. 2023). In mice, taurine supplementation was found to reverse age‐related tissue damage, suggesting its potential benefits for tissue repair (Rinaldi et al. 2024). Research has also demonstrated that taurine exerts protective effects against structural and functional abnormalities in offspring kidneys caused by maternal chronic kidney disease (Tain et al. 2023). Taurine has been demonstrated to extend lifespan in animals and improve multiple age‐related physiological functions, such as reducing cellular senescence, preventing telomerase deficiency, inhibiting mitochondrial dysfunction, reducing DNA damage, and alleviating inflammation (Izquierdo 2024; Thukral et al. 2025; Yang et al. 2012). Our study demonstrated that PCA normalized the expression of CSAD, a key enzyme involved in taurine biosynthesis, consistent with transcriptomic predictions. By restoring taurine metabolism, PCA may enhance antioxidant defense, reduce inflammation, and improve mitochondrial function, thereby conferring renal protection. These results highlight taurine metabolism as a novel target in delaying kidney aging and support PCA as a natural modulator of this pathway. Considering that taurine biosynthesis is closely linked to sulfur‐containing amino acid metabolism and cellular redox balance (Seidel et al. 2019), the normalization of CSAD expression observed in the present study may reflect an improvement in metabolic homeostasis under PCA treatment. Enhanced regulation of cysteine utilization and oxidative stress status may facilitate taurine synthesis and contribute to the restoration of taurine and hypotaurine metabolism. In addition, improved mitochondrial function and reduced inflammatory burden may further support the maintenance of taurine metabolic pathways. Although these possibilities require further experimental validation, they provide a plausible explanation for how PCA may indirectly regulate taurine metabolism and thereby contribute to renal protection in aging. Importantly, PCA has also been reported to exert protective effects in multiple organs, including the brain, liver, and cardiovascular system, primarily through its antioxidant and anti‐inflammatory activities (Song et al. 2020); therefore, the present findings extend these observations and suggest that modulation of taurine metabolism may represent a kidney‐related mechanism contributing to its anti‐aging effects.

In summary, the present study provides comprehensive evidence that PCA alleviates D‐galactose‐induced renal aging in mice through multiple mechanisms, including antioxidative and anti‐inflammatory effects, as well as the improvement of renal histopathological damage. Notably, multi‐omics integration analysis suggested that taurine and hypotaurine metabolism may represent a key metabolic pathway mediating these protective effects. These findings not only support PCA as a potential intervention strategy for delaying renal senescence but also place its renoprotective effects within the broader context of its multi‐organ biological activities.

Nevertheless, several limitations of this study should be acknowledged. First, the D‐galactose‐induced aging model used in this study primarily reflects accelerated aging driven by persistent oxidative stress rather than naturally occurring renal aging. Therefore, this model may only partially mimic physiological aging and could limit the extrapolation of our findings to natural aging or clinical conditions. Second, only healthy male BALB/c mice were included in the present study, while female animals were not evaluated. Consequently, potential sex‐specific effects of PCA and taurine‐related metabolic regulation remain unclear. Previous studies have indicated that sex hormones may influence oxidative stress responses and taurine metabolism (Seghieri et al. 2025), suggesting that the renoprotective effects of PCA in female animals warrant further investigation. Third, although multi‐omics integration identified taurine and hypotaurine metabolism as an important pathway associated with the renoprotective effects of PCA, the current evidence is primarily based on correlative analyses. Direct functional validation, such as taurine depletion or pathway inhibition experiments, is still required to establish the causal role of this metabolic pathway in mediating PCA‐induced protection. Finally, this study did not include comparisons between PCA and established nephroprotective agents; therefore, its relative efficacy and potential clinical advantages remain uncertain. As a result, the translational relevance of PCA for human renal aging still requires further investigation.

Future studies should validate the protective effects of PCA in naturally aged models or disease models that more closely resemble clinical conditions. In addition, functional experiments are needed to further elucidate the regulatory role of taurine metabolism in the anti‐aging effects of PCA. Moreover, preclinical and clinical studies will be necessary to evaluate the therapeutic potential of PCA in delaying renal aging and to explore its possible synergistic effects with other natural compounds or therapeutic strategies.

## 5 Conclusion

In conclusion, this study demonstrates that PCA effectively mitigates D‐galactose‐induced renal aging in mice. Integrative multi‐omics analyses identified taurine and hypotaurine metabolism as a key pathway potentially underlying the renoprotective effects of PCA. These findings highlight the metabolic basis of PCA‐mediated protection against kidney senescence and provide new insights into the regulation of age‐related renal dysfunction. Overall, our results suggest that PCA may represent a promising natural candidate for interventions targeting renal aging, although further studies are required to evaluate its translational and clinical potential.

## Author Contributions

**Feng Gao:** methodology, validation, investigation, software, data curation. **Lulu Xu:** validation, visualization, investigation, methodology, conceptualization. **Songhao Tian:** conceptualization, methodology, writing – review and editing, project administration, resources, software, writing – original draft, data curation. **Tao Chen:** validation, project administration, resources, investigation. **Zhihua Zhao:** investigation, methodology. **Yuyao Du:** visualization, investigation, methodology. **Jiarui Zhao:** conceptualization, methodology, software.

## Funding

This work was supported by Lvliang City Science and Technology Plan Project (2025SHFZ48), Doctoral Research Initiation Grant of Fenyang College, Shanxi Medical University (2024BS09), Science and Technology Innovation Program for Higher Education Institutions in Shanxi Province (2025L190), the Innovation and Entrepreneurship Training Program for University Students in Shanxi Province (20252026).

## Conflicts of Interest

The authors declare no conflicts of interest.

## Data Availability

The data presented in this study are available upon request from the corresponding author.
